# The Role of Oxymatrine in Amelioration of Acute Lung Injury Subjected to Myocardial I/R by Inhibiting Endoplasmic Reticulum Stress in Diabetic Rats

**DOI:** 10.1155/2020/8836904

**Published:** 2020-11-26

**Authors:** Yongpan Huang, Xian Long, Xinliang Li, Saihua Li, Jianbin He

**Affiliations:** ^1^Medicine School, Changsha Social Work College, Changsha, Hunan, China; ^2^Institute of Chinese Meteria Medica, Hunan Academy of Chinese Medicine, Changsha, China; ^3^Nursing Department, Guilin People's Hospital, Guilin, China; ^4^Department of Respiratory and Critical Care Medicine, The First People's Hospital of Huaihua, affiliated to University of South China, Huaihua, Hunan, China

## Abstract

**Background:**

Oxymatrine (OMT) is the primary pharmacological component of *Sophora flavescens* Aiton., which has been shown to possess potent antifibrotic, antioxidant, and anti-inflammatory activities. The aim of the present study was to clarify the protective mechanism of OMT on acute lung injury (ALI) subjected to myocardial ischemia/reperfusion (I/R).

**Methods:**

A myocardial I/R-induced ALI model was achieved in diabetic rats by occluding the left anterior descending coronary artery for 1 h, followed by reperfusion for 1 h. The levels of inflammatory factors (tumor necrosis factor-*α*, interleukin- (IL-) 6, and IL-17) in bronchoalveolar lavage fluid were assessed using commercially available kits. The index of myocardial injury, including the detection of cardiac troponin I (cTnI), cardiac troponin T (cTnT), lactate dehydrogenase (LDH), and creatine kinase-MB (CK-MB), was also determined using commercially available kits. Hematoxylin and eosin staining and terminal deoxynucleotidyl transferase-mediated dUTP nick end labeling were used to identify histological changes. The expression levels of endoplasmic reticulum chaperone BiP (GRP78), DNA damage-inducible transcript 3 protein (CHOP), eukaryotic translation initiation factor 2-alpha kinase 3 (PERK), inositol dependent enzyme 1*α* (IRE1*α*), ATF6, caspase-3, -9, and-12, Bcl-2, and Bax were determined by Western blotting. The mRNA expression levels of GRP78 and CHOP were detected by reverse transcription-quantitative PCR.

**Results:**

Myocardial I/R increased the levels of cTnI, cTnT, LDH, and CK-MB in diabetic rats. Damaged and irregularly arranged myocardial cells were also observed, as well as more serious ALI with higher lung injury scores and WET/DRY ratios and lower PaO_2_. Moreover, the expression of key proteins of endoplasmic reticulum stress (ERS) was increased by I/R injury, including phosphorylated- (p-) PERK, p-IRE1ɑ, and ATF6, as well as decreased levels of apoptosis. These effects were all significantly reversed by OMT treatment.

**Conclusions:**

OMT protects against ALI subjected to myocardial I/R by inhibiting ERS in diabetic rats.

## 1. Introduction

Diabetes is a metabolic disease with a high rate of mortality, which is characterized by hyperglycemia. Persistent hyperglycemia can lead to chronic tissue damage, including cardiomyopathy, nephropathy, diabetic foot, and diabetic neuropathy [1, 2]. Acute myocardial infarction is associated with higher mortality rates in patients with diabetes [3]. Diabetes also increases susceptibility myocardial ischemia/reperfusion (I/R) injury, which can induce endoplasmic reticulum stress (ERS) [4, 5]. ERS refers to ischemia, hypoxia, oxidative stress, and glucose/nutrition abnormalities where material deficiencies promote abnormal glycosylation reactions and calcium ion homeostasis, significantly increasing the number of unfolded proteins in the ER. As such, the processing capacity of the ER is exceeded causing abnormal cellular reactions, and the intracellular conditions are altered in an attempt to restore the ER environment. Moderate ERS restores homeostasis and maintains cell survival, but persistently severe ERS results in apoptosis and even necrosis, accelerating I/R injury. Therefore, suppressing ERS may become an important therapeutic option for ameliorating I/R injury [6–8].

Oxymatrine (OMT) is a major active ingredient isolated from *Sophora flavescens* Aiton., which possesses diverse biological properties beneficial to human health, including anti-inflammatory, antiallergic, antiviral, and antifibrotic activities. Owing to its antioxidant activity, OMT has been reported to exert protective effects against cardiovascular diseases [9], diabetes [10], inflammation [11, 12], and cancer [13, 14]. Several lines of evidence have demonstrated that OMT exerts its protective effects by scavenging lipid free radicals, thereby decreasing cytotoxicity in vitro and in vivo [15, 16]. Studies have also revealed that OMT ameliorates diabetes-associated aortic endothelial dysfunction in streptozotocin- (STZ-) induced diabetic mice [10, 17, 18]. Based on the role of apoptosis during ERS and the effect of OMT in myocardial I/R-induced ALI in diabetic rats, the current study aimed to further investigate the protective effects of OMT in this setting and to confirm the contribution of ERS-associated signaling pathways against ALI-induced apoptosis in diabetic rats. The results suggest that the efficacy of OMT in ALI is associated with the attenuation of ERS-induced apoptosis, which provides novel insights for the treatment of myocardial I/R-induced ALI.

## 2. Materials and Methods

### 2.1. Induction and Assessment of Diabetes

Sprague–Dawley rats weighing 180–200 g (8–10 weeks old) were provided by the Department of Experimental Animal Science, Xiangya School of Medicine, Central South University. All animal experiments were approved by the Hunan Academy of Chinese Medicine Animal Care and Use Committee (approval no. Xiang 2019-0013) and conducted in accordance with the United States National Institutes of Health Guide for the Care and Use of Laboratory Animals (NIH Publication No. 85–23, revised 1996) and Ethics Committees in Science: European Perspectives (19). The rats were fed a normal diet of standard feed for 1 week and then randomly divided into the control (*n* = 10) and model (*n* = 60) groups. The control group continued to receive the normal diet, and the model group received a high-sugar, high-fat diet (67.5% standard feed, 10% lard, 20% sucrose 20%, 2% cholesterol, and 0.5% pig bile salt). After 4 weeks, the model group (with diabetes mellitus (DM)) received a single intraperitoneal injection of STZ (Sigma-Aldrich: 572201) (35 mg/kg) and the high-sugar, high-fat diet was continued; for the control group (control), an equal volume of sodium citrate buffer was administered (in place if STZ), and the normal diet was continued. At the 8th week, blood was taken from the tail vein, and the glucose concentration was determined; fasting blood glucose ≥7.0 mmol/l or random blood glucose ≥11.0 mmol/l was considered to indicate diabetes. The rats were then intragastrically administered with saline or OMT (Aladdin: A111285) for 7 weeks and then intraperitoneally injected with normal saline.

### 2.2. Establishment of Myocardial I/R Injury

The coronary artery ligation method was used to establish the I/R injury model. After anesthetization with an intraperitoneal injection of sodium pentobarbital (30 mg/kg), the rats received endotracheal intubation and artificial ventilation with oxygen-enriched room air using a rodent respirator (Chengdu Techman Software Co., Ltd) with 60 breaths per minute, and the tidal volume was set to 8 ml. The heart was exposed, and thread was passed through the left coronary artery. Another 2 threads were drawn from the knot to loosen the ligature. The left coronary artery was ligated to produce ischemia, after which the local myocardium appeared cyanotic. After 1 h ischemia, the ligature was loosened to restore blood flow and initiate reperfusion, which was sustained for an additional hour.

**Figure 1:**
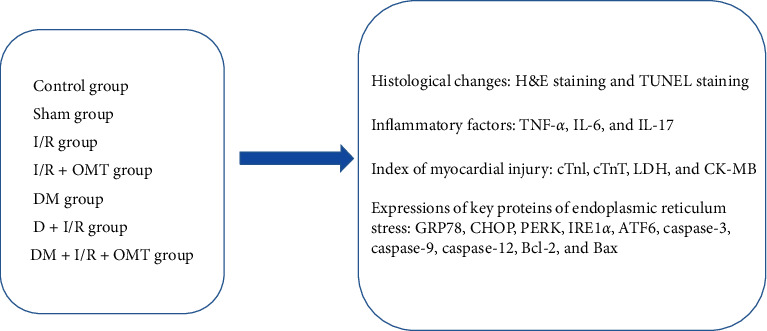
Experimental protocols.

The rats were randomly divided into the seven following (*n* = 8 per group) groups: (i) control group; (ii) Sham group, surgery with no ischemia; (iii) I/R group, myocardial I/R; (iv) I/R + OMT group, myocardial I/R + OMT 30 mg/kg; (v) diabetes mellitus (DM) group; (vi) DM + I/R group, DM + myocardial I/R; and (vii) DM + I/R + OMT group, DM + myocardial I/R + OMT 30 mg/kg. OMT (30 mg/kg) was chosen from our preliminary pilot experiment and dissolved in isotonic saline and administered by gavage 10 min prior to occlusion of the left coronary artery. During the experiment, about two animals per group died. The rate of mortality reached to 20%, which is acceptable based on the ethical approval granted to our study. At the end of the study, rats were anesthetized by an intraperitoneal injection of pentobarbital sodium (30 mg/kg). Blood samples were collected from abdominal aorta for subsequent analysis. All the animals were sacrificed following anesthesia by exsanguination, and their heart tissues were collected for the following experiments (Figure 1).

### 2.3. Detection of Serum Myocardial Enzyme Levels

At the end of the 8^th^ week, the rats were fasted for 12 h, sacrificed, and blood samples were collected. The serum was separated by centrifugation at 3000 × g for 10 min (4˚C), and the levels of cardiac troponin I (cTnI) (Sigma-Aldrich: MABX7161), cardiac troponin T (cTnT) (Sigma-Aldrich: SAB1402377), lactate dehydrogenase (LDH) (Sigma-Aldrich: L7016), and creatine kinase-MB (CK-MB) (Sigma-Aldrich: MAK116) were quantified using commercial ELISA kits. The remaining serum was stored at −80˚C for further studies.

### 2.4. Blood Gas Analysis and WET/DRY Ratio

Immediately after 1 h of reperfusion, the chest of each rat was opened, and arterial blood was taken from the left ventricle. Blood gas analysis was performed using the i-STAT blood gas analyzer (Abbott). Fresh lung tissues were weighed (wet weight) and dehydrated in an oven at 65˚C for 48 h. The tissues were then weighed again until the weight did not fluctuate for ≥3 measurements. The ratio of wet weight to dry weight (the WET/DRY ratio) was then calculated.

### 2.5. Collection of Bronchoalveolar Lavage (BAL) Fluid

After sacrifice, the lungs were removed from each rat. The tissues were carefully separated, the left bronchial hilar was ligated, and scissors were used to cut a *V*-shaped incision under the tracheal annular cartilage. A blunt needle was inserted into the trachea along the incision, and the needle was ligated and fixed with a thin wire. Lung tissues were lavaged 3 times with PBS to collect BAL fluid. The collected samples were immediately centrifuged at 3000 × g for 10 min (4˚C), and the supernatant was stored at −80˚C until subsequent experimental testing.

### 2.6. Detection of Leukocytes, Tumor Necrosis Factor-*α* (TNF-*α*), Interleukin- (IL-) 6, and IL-17 in BAL Fluid

The obtained BAL fluid was centrifuged at 300 × g for 5 min (4˚C), and the resulting supernatant was collected for protein concentration analysis; the cell pellet was stored for further use. Briefly, 6–10 times the amount of red blood cell lysis buffer (Roche Diagnostics: 11814389001) was added to the cell pellet, and the cells were incubated on ice for 5 min. Ice-cold PBS was added to the cell lysate, which was then centrifuged at 500 × g for 5 min (4˚C). The supernatant was discarded, and the cells were centrifuged in 1 ml PBS (500 × g for 5 min, 4˚C) to remove any remaining red blood cell debris. The supernatant was discarded, and the cells were resuspended in 0.5 ml PBS and counted using a hemocytometer. The levels of inflammatory factors were determined using commercial ELISA kits (Sigma-Aldrich: T5944, RAB0311, SRP6511), and all experiments were performed according to the manufacturer's instructions.

### 2.7. Hematoxylin and Eosin (H&E) Staining

Tissues of the left lung and myocardial tissues of the left ventricle were collected, fixed in 4% paraformaldehyde for 24 h, and then dehydrated until transparent. The tissues were embedded in paraffin and sectioned (4 *μ*m), and H&E staining was conducted. The tissues were observed in 3 random areas under a light microscope (200x). Inflammatory cell infiltration, hemorrhage, and the interstitial and alveolar septum thickness of 5 random fields were observed, and scores for the corresponding indicators of lung injury were determined. For inflammatory cell infiltration (i) 0, without damage; (ii) 1, mild injury; (iii) 2, moderate injury; (iv) 3, severe damage; and (v) 4, very severe tissue damage. Other lung injury index scores were also calculated, and the average scores for each treatment group were determined.

### 2.8. Detection of Apoptosis

Tissue apoptosis was determined by terminal deoxynucleotidyl transferase-mediated dUTP nick end labeling (TUNEL). Briefly, at the end of reperfusion, the lungs were excised and fixed in 4% paraformaldehyde in PBS at room temperature for 24 h. Fixed tissues were embedded in paraffin and stained using an in situ cell death detection kit (Roche Diagnostics: 11684795910) as per the manufacturers' protocol. The apoptotic index (percentage of TUNEL-positive nuclei/total number of nuclei) was then determined.

### 2.9. Detection of Caspase-3 Activity

Tissues were collected and rinsed with PBS, homogenized and lysed as aforementioned, and then centrifuged at 12,000 × g for 10 min (4˚C). Caspase-3 activity was detected using a caspase 3 assay kit (Roche Diagnostics: CASP3C) and normalized to the total protein content for quantification.

### 2.10. Western Blot Analysis

Total protein was extracted from the lung tissues and transferred to PVDF membranes by electrophoresis (SDS-PAGE). The membranes were incubated with primary antibodies for 2 h at room temperature, rinsed, and subsequently incubated with a horseradish peroxidase-conjugated secondary antibody. The chemiluminescence signals were detected using the EasySee Western Blot Kit (Beijing TransGen Biotech Co., Ltd.), and densitometric analysis was conducted with ImageJ Software 1.43 (National Institutes of Health). Primary antibodies against the following targets were used: endoplasmic reticulum chaperone BiP (GRP78) (1 : 1000, G8918 : Sigma-Aldrich; Merck KGaA), DNA damage-inducible transcript 3 protein (CHOP) (1 : 1000, SAB4500631 : Sigma-Aldrich; Merck KGaA), inositol dependent enzyme 1 (IRE1)*α* (1 : 1000, P4334 : Sigma-Aldrich; Merck KGaA), p-IRE1*α* (1 : 1000, 5.32758: Sigma-Aldrich; Merck KGaA), eukaryotic translation initiation factor 2-alpha kinase 3 (PERK) (1 : 1000, P0073 : Sigma-Aldrich; Merck KGaA), p-PERK (1 : 1000, SAB4301310 : Sigma-Aldrich; Merck KGaA), ATF6 (1 : 1000, PRS3681 : Sigma-Aldrich; Merck KGaA), caspase-9 (1 : 1000, SAB4300683 : Sigma-Aldrich; Merck KGaA), caspase-3 (1 : 1000, C9598 : Sigma-Aldrich; Merck KGaA), caspase-12 (1 : 1000, C7611 : Sigma-Aldrich; Merck KGaA), Bax (1 : 1000, B8429 : Sigma-Aldrich; Merck KGaA), Bcl-2 (1 : 1000, B3170 : Sigma-Aldrich; Merck KGaA), and GADPH (1 : 1000, AF0006; Beyotime Biotechnology, China).

### 2.11. Real-Time PCR

Gene expression levels (mRNA) in lung tissue were determined by real-time PCR (Applied Biosystems). First, extracted total RNA were purified with 75% ethanol, and its concentration was determined by spectrophotometry. Then, the purified total RNA (200 ng each sample) was added into a transcription kit (DRR037A; TaKaRa, Dalian, China) and mixed to get the first strand template (reverse transcription reaction). GAPDH was used as the loading control. The primers were provided by GeneCopoeia Inc., as follows: CHOP forward, 5′-CAT ACA CCA CCA CAC CTG AAA G-3′; reverse, 5′-CAT ACA CCA CCA CAC CTG AAA G-3′; GRP78 forward, 5′-TCT CCA CGG CTT CCG ATA AT-3′; and reverse, 5′-GTA CCT TTG TCT TCA GCT GTC ACT C-3′. All oligonucleotide primers were designed by Sangon Biotech Co., Ltd (Shanghai, China). The Ct value of the target is normalized by subtracting the GAPDH Ct value to provide a ΔCt value. The relative expression level between treatments was then calculated using the following equation: relative gene expression = 2 − (ΔCt sample −ΔCt control).

### 2.12. Statistical Analysis

SPSS 21.0 (IBM Corp.) was used for statistical analysis, and all data are presented as the mean ± standard deviation (SD). For gene expressions, one-way ANOVA with the post hoc Tukey test was used to test for differences among groups. Lung injury scores were analyzed with the Kruskal–Wallis test followed by Dunn's multiple comparison test. The statistical significance of differences was assessed at *P* < 0.05.

## 3. Results

### 3.1. Myocardial I/R Accelerates ALI in Diabetic Rats

**Figure 2:**
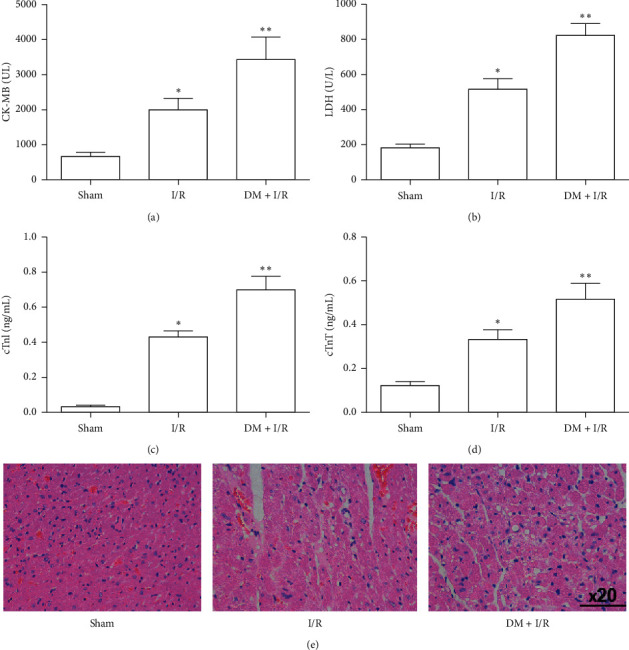
Myocardial I/R-accelerated acute lung injury in diabetic rats. (a), (b), (c), and (d) Serum levels of CK-MB, LDH, cTnl, and cTnT. (e) Hematoxylin and eosin staining of cardiac tissues (original magnification, 400x). Bars represent the mean ± SD of three independent experiments. ^*∗*^*P* < 0.05 vs. the Sham group; ^*∗∗*^*P* < 0.01 vs. the I/R group. I/R, ischemia/reperfusion; CK-MB, creatine kinase-MB; LDH, lactate dehydrogenase; cTnI, cardiac troponin I; cTnT, cardiac troponin T.

After the 8^th^ week, blood was taken from the tail vein, and the glucose concentration was determined; fasting blood glucose ≥7.0 mmol/l or random blood glucose ≥11.0 mmol/l was considered to indicate diabetes (Supplementary Figure (available here)). To investigate the protective effect of OMT against I/R-induced myocardial damage in diabetic rats, alterations in the concentrations of serum myocardial injury markers (cTnI, cTnT, LDH, and CK-MB) were observed. The levels of serum cTnI, cTnT, LDH, and CK-MB were significantly increased in diabetic rats with I/R, compared with those in the Sham group. However, OMT treatment markedly suppressed the increases in these serum constituents (Figure 2). In addition, OMT ameliorated the damage and irregular arrangement of myocardial cells induced by I/R injury (Figure 2). These findings suggest that OMT attenuates myocardial I/R injury in diabetic rats.

**Figure 3:**
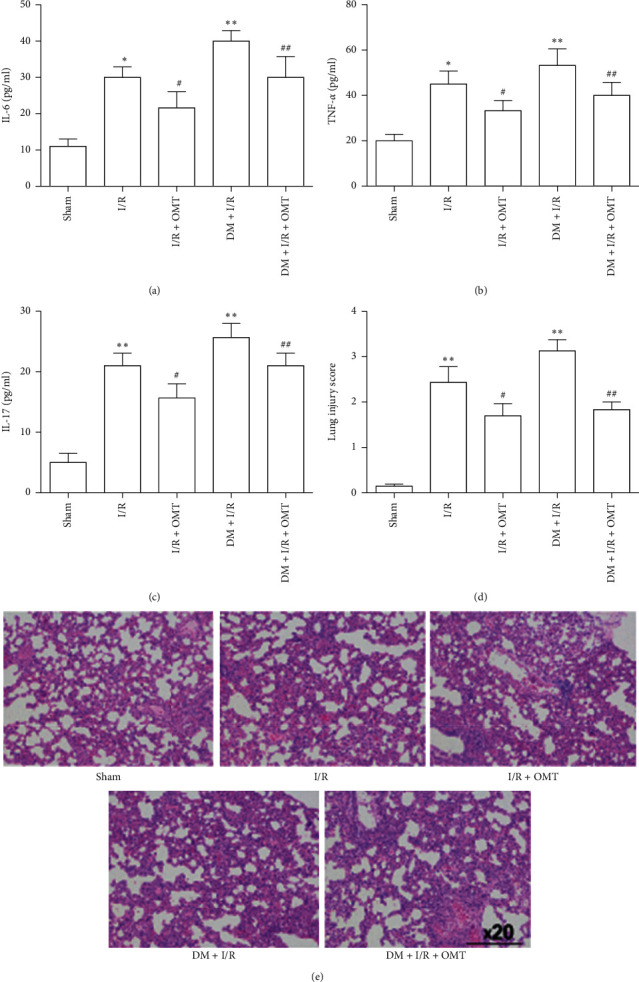
OMT ameliorates myocardial I/R-induced acute lung injury in diabetic rats. (a), (b), and (c) Expression levels of inflammatory factors IL-6, TNF-*α,* and IL-17. (d) Severity of lung injury expressed as the injury score. (e) Histopathological changes in rat lung tissues from control and diabetic rats subjected to sham or myocardial I/R surgery (original magnification, x200). All values are expressed as the mean ± SD, *n* = 8. ^*∗*^*P* < 0.05 vs. the Sham group; ^#^*P* < 0.05 vs. the I/R group; ^##^*P* < 0.01 vs. the DM + I/R group. OMT, oxymatrine; I/R, ischemia/reperfusion; IL, interleukin; TNF-*α*, tumor necrosis factor-*α*; DM, diabetes mellitus.

ALI secondary to myocardial I/R is reportedly aggravated by diabetes [19, 20]. Following myocardial I/R, the BAL fluid was collected and analyzed. As indicated in Figure 3, the levels of TNF-*α*, IL-6, and IL-17A in the BAL fluid were significantly increased in diabetic rats, indicating the occurrence of a severe inflammatory reaction following myocardial I/R insult. Furthermore, OMT treatment suppressed these increases in TNF-*α*, IL-6, and IL-17A. The severity of lung injury was assessed by H&E staining and graded using an injury scoring system. As shown in Figure 3, H&E staining of the lung tissue revealed ALI with alveolar and interstitial edema, hemorrhage, and inflammatory cell infiltration. These pathological changes were more severe in diabetic rats. Compared with the Sham group, the degree of lung injury in the myocardial I/R group was also increased. These results confirmed that myocardial I/R could deteriorate ALI in the diabetic rats. However, OMT ameliorated myocardial I/R-induced ALI, suggesting a protective affect against ALI secondary to myocardial I/R in diabetic rats.

### 3.2. OMT Inhibits ALI-Induced ERS in Diabetic Rats

**Figure 4:**
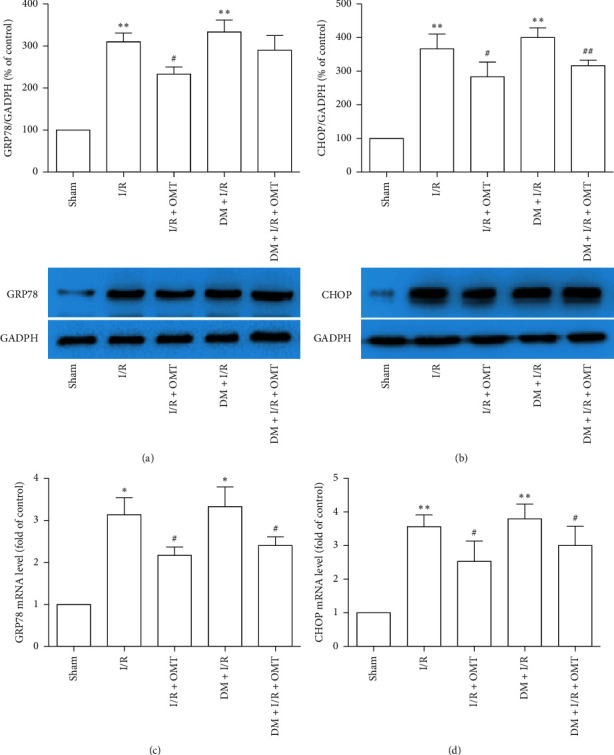
Effects of OMT on endoplasmic reticulum stress during myocardial I/R-induced acute lung injury in diabetic rats. mRNA expression levels of (a) GRP78 and (b) CHOP were detected using Western blot analysis. Protein expression levels of (c) GRP78 and (d) CHOP were determined using reverse transcription-quantitative PCR. Values are expressed as the mean ± SD from three independent experiments, *n* = 3. ^*∗*^*P* < 0.05 and ^*∗∗*^*P* < 0.01 vs. the Sham group; ^##^*P* < 0.01 vs. the I/R group; ^##^*P* < 0.01, vs. the DM + I/R group. OMT, oxymatrine; I/R, ischemia/reperfusion; GRP78, endoplasmic reticulum chaperone BiP; CHOP, DNA damage-inducible transcript 3 protein; DM, diabetes mellitus.

An increasing number of studies have suggested that excessive ERS accelerates the severity of I/R injury [21, 22]. To determine whether OMT treatment exerts a protective effect against myocardial I/R-associated ALI by inhibiting ERS, the expression levels of ERS markers GRP78 and CHOP were detected in diabetic rats. As shown in Figure 4, the expression of GRP78 and CHOP was markedly increased in the diabetic rats compared with the Sham group. Consistent with protein expression, the levels of GRP78 and CHOP mRNA were assessed by reverse transcription-quantitative PCR. We found that GRP78 and CHOP mRNA expressions are consistent with the protein expressions. Further findings showed no obvious differences between the I/R group and the DM + I/R group in the current experimental background, which does not mean that no differences in injury occurred between the two groups. Treatment with OMT downregulated GRP78 and CHOP mRNA expressions in myocardial I/R-associated ALI. These results suggest that OMT treatment alleviates ERS resulting from myocardial I/R-associated ALI in diabetic rats.

### 3.3. OMT Inhibits ERS-Induced Apoptosis in Diabetic Rats

**Figure 5:**
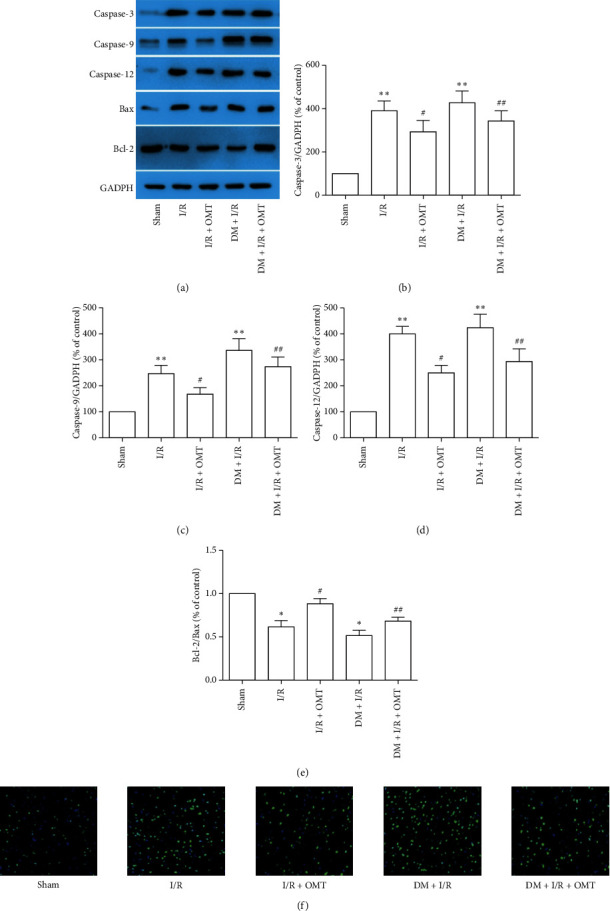
OMT inhibits ERS-induced apoptosis in diabetic rats. (a) Expression of caspase-3, caspase-9, caspase-12, Bax, and Bcl-2 was determined using Western blot analysis. (b), (c), (d), and (e) Quantification of Western blot results. (f) TUNNEL staining in lung tissues. Values are expressed as the mean ± SD from three independent experiments, *n* = 3. ^*∗*^*P* < 0.05 and ^*∗∗*^*P* < 0.01 vs. the Sham group; ^##^*P* < 0.01 vs. the I/R group; ^##^*P* < 0.01, vs. the DM + I/R group. OMT, oxymatrine; ERS, endoplasmic reticulum stress; I/R, ischemia/reperfusion; DM, diabetes mellitus.

To confirm whether ALI-induced ERS is involved in apoptosis, Western blotting was used to determine the expressions of apoptosis-related proteins, including caspase-9, caspase-3, caspase-12, Bcl-2, and Bax, in the lung tissues of diabetic rats. ALI caused significant increases in caspase-9, caspase-3, caspase-12, and Bax and a decrease in Bcl-2 expression (Figure 5), and OMT treatment reversed these expression patterns. These results demonstrate that OMT inhibits ERS-induced apoptosis in rats including diabetes and nondiabetes. However, the present study does not provide any evidence for diabetes-specific effects.

### 3.4. OMT Exhibits Protective Effects against ALI-Induced ERS-Associated Signaling Pathway Activation in Diabetic Rats

**Figure 6:**
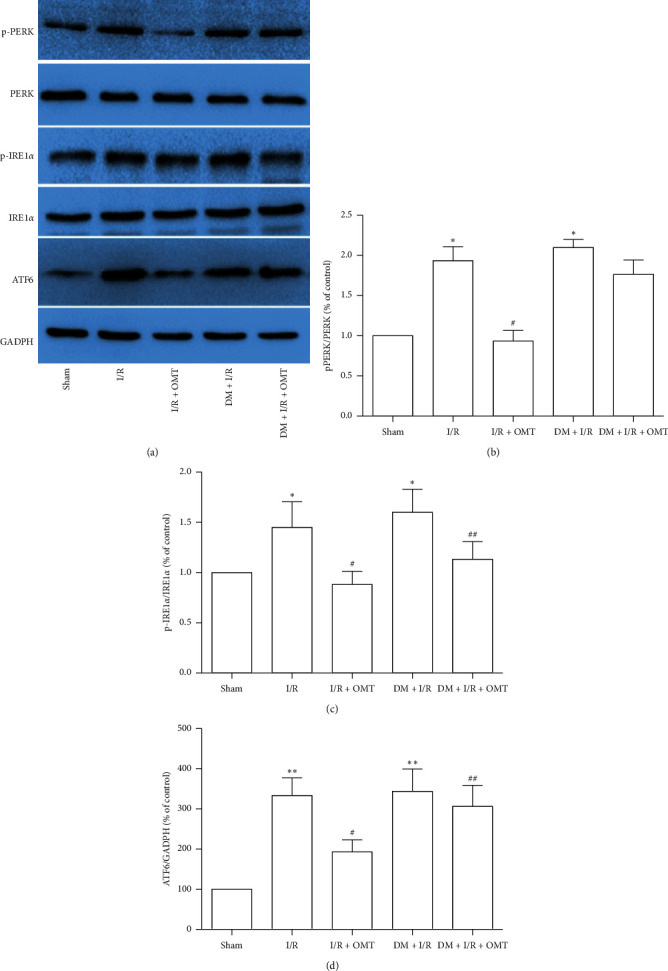
OMT inhibits acute lung injury-induced endoplasmic reticulum stress-associated signaling pathway activation in diabetic rats. (a) Protein expression levels of p-PERK, PERK, p-IRE1*α*, and IRE1*α* were determined using Western blot analysis. (b), (c), and (d) Quantification of Western blot results. Values are expressed as the mean ± SD from three independent experiments, *n* = 3. ^*∗*^*P* < 0.05 and ^*∗∗*^*P* < 0.01 vs. the Sham group; ^#^*P* < 0.05 vs. the I/R group; ^##^*P* < 0.01, vs. the DM + I/R group. OMT, oxymatrine; PERK, translation initiation factor 2-alpha kinase 3; IRE1, inositol dependent enzyme 1; p-, phosphorylated; I/R, ischemia/reperfusion; DM, diabetes mellitus.

Based on the aforementioned results, the mechanisms of OMT against myocardial I/R-induced ALI, which were associated with ERS-associated signaling proteins such as IRE1*α*, PERK, and ATF6, were investigated. As shown in Figure 6, ALI significantly upregulated the expressions of p-IRE1*α* and p-PERK, compared with the Sham group. Furthermore, these changes were reversed by OMT. These observations indicate that OMT inhibits IRE1*α* and/or PERK pathways, thereby attenuating ERS-mediated apoptosis and eliciting protection in ALI secondary to myocardial I/R.

## 4. Discussion

Myocardial I/R injury is regarded as a major public health threat with high rates of morbidity and mortality. Extensive studies have demonstrated that myocardial I/R may result in distant organ damage and that the lung may be one of the most vulnerable organs. The primary novelty of the present study was presenting evidence that myocardial I/R-induced ALI induces excessive lung ERS in diabetic rats and that OMT administration inhibits ERS-induced apoptosis. The myocardial protective effects of OMT suggest that it may be an effective agent for the treatment of I/R injury. However, recent studies showed that ER stress-induced apoptosis plays an important role in myocardial I/R-induced ALI. However, the more accurate functions have not been fully yet elucidated. In present study, the absence of DM without the I/R injury group might hinder understanding of myocardial I/R-induced ALI-associated ERS and the protective of OMT.

OMT is a major active ingredient isolated from *Sophora flavescens* Aiton. Several lines of evidence have demonstrated that OMT possesses diverse pharmacological characteristics, such as anti-inflammatory, antiallergic, antiviral, and antifibrotic properties [10, 11, 15–18]. OMT has been widely applied for the prevention and treatment of liver pathologies, cardiovascular diseases, vascular injury, and diabetes-associated dysfunction and inflammation [10, 15, 17]. In spite of these findings, the protective effects of OMT, as well as its potential mechanism, are yet to be elucidated. As such, the present study aimed to investigate the protective effects of OMT on myocardial I/R-associated ALI in diabetic rats.

Diabetes is the most common of all endocrine diseases. Diabetes is characterized by persistent hyperglycemia, which may lead to diverse representative complications including cardiomyopathy, nephropathy, diabetic foot, and diabetic neuropathy, which significantly contribute to the associated rates of morbidity and mortality [2]. Additionally, diabetes may accelerate the deterioration of respiratory function with characteristic anatomical and biological changes to the diabetic lung [5, 23, 24]. These abnormalities affect lung volume, pulmonary diffusing capacity, ventilation control, bronchomotor tone, and neuroadrenergic bronchial innervation. Although the practical implications of these functional alterations are frequently disregarded, the presence of an associated acute or chronic pulmonary and/or cardiac attack could influence severe respiratory derangement in diabetes [25]. During myocardial ischemia and/or reperfusion, the membranes of the myocardial tissues are attacked, resulting in the release of myocardial enzymes, including cTnI, cTnT, LDH, and CK-MB (which are often regarded as the markers of myocardial injury), into the peripheral blood. Therefore, detecting the peripheral blood levels of these enzymes (which reflect the degree of myocardial injury) has proven considerably valuable in the diagnosis of myocardial infarction. According to the results of the present study, OMT notably decreases the serum concentrations of these enzymes. Additionally, pathomorphological studies indicated a reduction in I/R-induced myocardial damage in diabetic rats treated with OMT. Consistent with previously reports [19], the present study revealed that the lungs of diabetic rats are susceptible to myocardial I/R injury, indicated by increased levels of ALI-induced inflammatory factors (including IL-6, TNF-*α*, and IL-17) with higher lung injury scores and WET/DRY ratios, as well as a lower PaO_2_. Moreover, the DM + I/R group showed more severe damages in levels of ALI-induced inflammatory factors (including IL-6, TNF-*α*, and IL-17) and lung injury scores but showed no obvious differences between the I/R + OMT group and the DM + I/R + OMT group. OMT could decrease the levels of inflammatory factors and lung injury scores and WET/DRY ratios in the I/R + OMT group or the DM + I/R + OMT group. But these results demonstrated that the effects of I/R + OMT is more obviously than DM + I/R + OMT. Overall, OMT exerts a beneficial effect on ALI secondary to I/R injury in diabetic rats.

The ER is a membrane-bound and structurally intricate organelle present in all eukaryotic cells. It is the specific site for monitoring intracellular protein and lipid synthesis, as well as intracytoplasmic calcium storage [26, 27]. Diabetes is known to be a chronic disorder characterized by low-grade chronic inflammation and a hypoxic microenvironment, which can result in the accumulation of misfolded and unfolded proteins in the ER lumen [4, 28]. ER homeostasis is disturbed in a condition referred to as ERS. Excessive ERS triggers an adaptive mechanism termed the unfolded protein response (UPR), which is implemented in the maintenance of cellular homeostasis. The UPR is primarily mediated by three ER transducers PERK, IRE1, and ATF6, which could detect unusual conditions and transmit signals to the cytosol. Once activated, these signals induced downstream responses. During ERS conditions, PERK, IRE1*α*, and ATF6 contributed to reactive oxygen species (ROS) generation and apoptosis. Sustained ERS ultimately leads to ERS-mediated apoptosis [9, 29, 30]. Caspase-12 is present in various tissues and is regarded as one of the major apoptotic signaling molecules of ERS. The imbalance of calcium ions in the ER or the accumulation of ER proteins can ultimately lead to caspase-12 expression. Excessive ERS can trigger the activation of other caspases, such as caspase-9 and -3, causing cascade reactions that ultimately result in cell death [27, 31]. CHOP is an important proapoptotic signaling molecule and ERS-specific transcription factor. Under physiological conditions, CHOP is expressed at low levels; once activated by ERS, its expression level significantly increases, and this is considered to be an important marker of ERS [31]. Intracellular apoptotic signaling usually activates the mitochondrial pathway, facilitating the release of mitochondrial proapoptotic proteins and apoptosis-inducing factors and ultimately activating the caspase cascade and promoting apoptosis [32]. The present study revealed that the expression of ERS marker proteins GRP78 and CHOP in the I/R group and the DM + I/R group were markedly upregulated, demonstrating that ERS is involved in the occurrence of myocardial I/R-induced ALI, but showed no obvious differences between these groups in the current experimental background, and there seems to be no significant difference between these groups, which does not mean that no differences in injury occurred between the two groups. Furthermore, the present study demonstrated that the protein expression levels of PERK, p-PERK, p-IRE1*α*, ATF6, and CHOP in the lung tissues were upregulated. These results suggested that the lung tissue damage in the DM + I/R group was more severe than that in the I/R group. Further study confirmed that OMT treatment inhibited the upregulation in p-PERK, p-IRE1*α,* and ATF6 and also reduced the apoptosis index, suggesting that OMT exerts protective effects against myocardial I/R-induced ALI by suppressing ERS-associated apoptosis. Collectively, these findings demonstrate that OMT inhibits ERS-associated signaling pathways, which may further decrease ERS-induced apoptosis. This was subsequently investigated by detecting the expressions of apoptosis-associated proteins. Notably, OMT downregulated the high expression levels of caspase-3, caspase-9, caspase-12, and Bax associated with myocardial I/R-induced ALI and upregulated Bcl-2 expression in diabetic rats.

## 5. Conclusions

In summary, the present study revealed the significant protective effects of OMT against myocardial I/R-induced ALI in diabetic rats and illustrated that these effects are mediated by the inhibition of ERS-associated apoptosis. However, the overall mechanisms underlying the protective effects of OMT and its association with ERS required further investigation.

## Data Availability

The data used to support the findings of this study are included within the manuscript and are available from the corresponding author upon request.

## Abbreviations

ALI: Acute lung injury
cTnI: Cardiac troponin I
cTnT: Cardiac troponin T
CK-MB: Creatine kinase-MB
CHOP: DNA damage-inducible transcript 3 protein
ERS: Endoplasmic reticulum stress
GRP78: Endoplasmic reticulum chaperone BiP
I/R: Ischemia/Reperfusion
IL-6: Interleukin- (IL-) 6
IRE1-*α*: Inositol dependent enzyme 1*α* (IRE1*α*)
LDH: Lactate dehydrogenase
OMT: Oxymatrine
p-PERK: Phosphorylated- (p-) PERK
PERK: Eukaryotic translation initiation factor 2-alpha kinase 3
STZ: Streptozocin
TUNEL: Terminal deoxynucleotidyl transferase-mediated dUTP nick end labeling.

## Supplementary Materials

Supplementary Materials Supplementary Figure1: effect of OMT on the serum glucose level. Bars represent the mean ± SD of three independent experiments. ^*∗*^*P* < 0.05 vs. the control group.
