# Supplementary figures and images for: The Role of Oxymatrine in Amelioration of Acute Lung Injury Subjected to Myocardial I/R by Inhibiting Endoplasmic Reticulum Stress in Diabetic Rats
Source: Evid Based Complement Alternat Med. 2020 Nov 26;2020:8836904. doi: 10.1155/2020/8836904 (PMC7714565; doi:10.1155/2020/8836904)

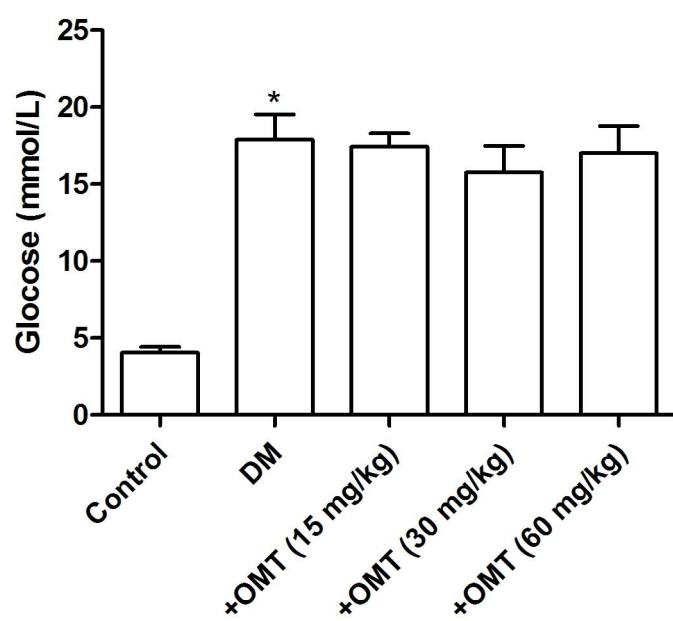

Supplementary figure 1

Supplement: Supplementary Materials — Supplementary Figure1: effect of OMT on the serum glucose level. Bars represent the mean ± SD of three independent experiments. ∗P < 0.05 vs. the control group. [file 8836904.f1.pdf]
